# ALEXA, ASSESS MY MEMORY: THE FEASIBILITY OF EXTENDED HEALTH MONITORING IN AN OLDER-ADULT-LIVING COMMUNITY

**DOI:** 10.1093/geroni/igz038.1224

**Published:** 2019-11-08

**Authors:** Jensen Davis, Shannon Howard, Gregory King, Phanidar Boddu, Kiran Jyothi, Joan McDowd

**Affiliations:** University of Missouri-Kansas City, Kansas City, Missouri, United States

## Abstract

The goal of most older adults is to live independently in their own homes, for as long as possible. There are many advantages to aging in place for the individual, but also challenges as changes in cognitive and physical health can occur over time. Especially for older adults living alone, tracking these changes is critical for early intervention and prevention. The relatively easy availability of consumer technology may provide one mechanism for monitoring older adults in their homes. We designed a pilot study to test the feasibility and acceptability of using wearable sensors (Fitbit sensors), in conjunction with automated interactive voice recognition technology (Amazon Echo), to monitor older adults’ physical and cognitive health during daily activities. Participants (7 females, 2 males; 65-80 years of age) were recruited from a housing complex for older adults with low income. They were interviewed about health monitoring technology before and after a 2-week measurement period during which they were expected to wear the Fitbit daily and interact with the Amazon Echo for 8 consecutive days. Feasibility challenges included limited skill in Echo interactions, remembering to do the assessments, and charging/uploading Fitbit data. Qualitative analysis of interviews revealed generally positive attitudes about technology, but low comfort operating the devices. These preliminary findings suggest that with additional training for older adults, sensors and voice recognition technologies could have significant roles in maintaining older adult quality of life by contributing to early detection of decline and timely intervention.

